# Deep Neural Networks for Automated Outer Plexiform Layer Subsidence Detection on Retinal OCT of Patients With Intermediate AMD

**DOI:** 10.1167/tvst.13.6.7

**Published:** 2024-06-14

**Authors:** Guilherme Aresta, Teresa Araujo, Gregor S. Reiter, Julia Mai, Sophie Riedl, Christoph Grechenig, Robyn H. Guymer, Zhichao Wu, Ursula Schmidt-Erfurth, Hrvoje Bogunovic

**Affiliations:** 1 Christian Doppler Laboratory for Artificial Intelligence in Retina, Department of Ophthalmology and Optometry, Medical University Vienna, Vienna, Austria; 2 Laboratory for Ophthalmic Image Analysis, Department of Ophthalmology and Optometry, Medical University of Vienna, Vienna, Austria; 3 Centre for Eye Research Australia, The Royal Victorian Eye and Ear Hospital, East Melbourne, VIC, Australia; 4 Department of Surgery (Ophthalmology), The University of Melbourne, Melbourne, VIC, Australia

**Keywords:** artificial intelligence, optical coherence tomography, age-related macular degeneration

## Abstract

**Purpose:**

The subsidence of the outer plexiform layer (OPL) is an important imaging biomarker on optical coherence tomography (OCT) associated with early outer retinal atrophy and a risk factor for progression to geographic atrophy in patients with intermediate age-related macular degeneration (AMD). Deep neural networks (DNNs) for OCT can support automated detection and localization of this biomarker.

**Methods:**

The method predicts potential OPL subsidence locations on retinal OCTs. A detection module (DM) infers bounding boxes around subsidences with a likelihood score, and a classification module (CM) assesses subsidence presence at the B-scan level. Overlapping boxes between B-scans are combined and scored by the product of the DM and CM predictions. The volume-wise score is the maximum prediction across all B-scans. One development and one independent external data set were used with 140 and 26 patients with AMD, respectively.

**Results:**

The system detected more than 85% of OPL subsidences with less than one false-positive (FP)/scan. The average area under the curve was 0.94 ± 0.03 for volume-level detection. Similar or better performance was achieved on the independent external data set.

**Conclusions:**

DNN systems can efficiently perform automated retinal layer subsidence detection in retinal OCT images. In particular, the proposed DNN system detects OPL subsidence with high sensitivity and a very limited number of FP detections.

**Translational Relevance:**

DNNs enable objective identification of early signs associated with high risk of progression to the atrophic late stage of AMD, ideally suited for screening and assessing the efficacy of the interventions aiming to slow disease progression.

## Introduction

New treatments for the atrophic form of age-related macular degeneration (AMD) are now a reality with the recent approvals of pegcetacoplan and avacincaptad pegol that have been shown to slow the growth of atrophic lesions.^1^^,^^2^ Many other novel interventions are also undergoing clinical trials, all currently aiming to intervene when geographic atrophy (GA) has already been identified. It has now become critical to identify people with the early/intermediate stages of AMD who are more at risk for progression to GA, to more specifically counsel them as to the changing landscape in their management, and to identify those who would potentially be candidates for earlier intervention once this becomes feasible.

Optical coherence tomography (OCT) has become the gold-standard imaging modality for the management of patients with AMD due to its ability to acquire cross-sectional images of the retina noninvasively and at a micrometer resolution. OCT is of particular value for providing imaging biomarkers characteristic of AMD and its progression such as the thickness of outer retinal layers, drusen volume, and hyperreflective foci.^3^ Recently, effort has been devoted to defining early atrophic lesions as seen on OCT, primarily through the Classification of Atrophy Meeting program, which defined incomplete and complete retinal pigment epithelial and outer retinal atrophy (iRORA, cRORA).^4^^,^^5^

The presence of subsidence of the inner nuclear layer (INL) and the outer plexiform layer (OPL), and/or hyporeflective wedge-shaped band(s) in Henle's nerve fiber layer (HFL), form a marker known as nascent GA (nGA).^6^ This marker has been reported to be a precursor of GA^6^ and a high-risk factor for its development.^7^^–^^9^ The retinal layer subsidence is expected to be one of the earliest robust precursors of GA. Correspondingly, Ferrara et al.,^8^ in a cohort of 80 patients with early/intermediate AMD, in which 40 progressed to advanced AMD and 40 did not, analyzed the biomarkers of neurosensory retina, photoreceptor layer, retinal pigment epithelium (RPE) abnormalities, features of nascent GA, and choroidal abnormalities and found OPL subsidence to be the most prognostic single OCT feature for progression to GA. This makes the identification of such retinal layer subsidences an important biomarker in people with intermediate AMD (iAMD). The detection of subsidence typically requires a comprehensive and detailed examination of individual B-scans in an entire cube. However, such manual interpretation of OCT images is both time-consuming and subjective, making it prone to interobserver variability.^10^ An automated way of identifying these important changes would facilitate the screening of subjects at high risk of progression and would be a useful biomarker in intervention trials aiming to slow progression.

The recent advances in artificial intelligence (AI), namely, deep learning, have shown potential for automating the detection of biomarkers. In retinal OCT, deep learning has already been used for the delineation of the retinal layers,^11^^,^^12^ as well as different pathologic manifestations.^13^^–^^17^ In particular, AI has shown potential for the detection of nGA features,^18^ but targeted AI-based automated subsidence detection and quantification is still an underexplored task. A typical use-case of deep learning systems is the automated annotation of pixels/voxels belonging to a given biomarker (i.e., biomarker segmentation), from which meaningful characteristics such as shape, area, or volume can be extracted. However, for some tasks, the fine pixel/voxel-wise segmentation of the biomarker of interest is either a nontrivial task or not relevant. In these cases, a proper alternative is to instead perform object detection, that is, characterize the lesions of interest by their central position (centroids) and bounding boxes. Deep learning methods for biomarker detection in medical images are commonly constructed in two steps.^19^ The first step consists of an initial candidate selection with high sensitivity, where the aim is to include all regions of interest likely containing the target candidate. This step is performed using an object detection framework such as YOLO, SSDNet, Faster R-CNN, or Mask R-CNN.^20^^–^^23^ Such detection, however, results in many false-positive (FP) detections. Thus, a second, separate model is trained and applied to recognize which of the candidates are indeed of interest (i.e., true-positive [TP] detections).

In this study, we propose and extensively evaluate a system for automated OPL subsidence detection in OCT volumes. In particular, we approach the problem as a volumetric object detection task on three-dimensional (3D) OCT scans. The regions found to be candidates for OPL subsidence are also used for predicting volume-, eye-, and subject-wise likelihood of the presence of this important biomarker. By indicating the location of possible lesions, the AI system has an inherent inexplicability, easing the assessment of the detection by the retinal expert and consequently its acceptance.

## Methods

### Data Collection and Annotation

#### Participants

Our method was developed and evaluated using the data from two longitudinal imaging studies of subjects with AMD, with eyes having drusen and no late AMD at baseline. The first data set corresponded to a subset of the participants randomized to the sham arm of the LEAD clinical trial (NCT01790802),^24^ who were seen at 6-monthly intervals up to a 3-year period. This subset included participants who did not have nGA (OPL and INL subsidence and/or a hyporeflective wedge-shaped band in Henle's nerve fiber layer) in either eye at baseline, based on independent masked grading of the OCT volume scans only as reported previously,^25^ and who had at least one follow-up visit. The LEAD data set thus contained OCTs from 280 eyes of 140 participants with bilateral large drusen AMD at baseline, where 77% of the participants were female and their mean age was 70 ± 8 years.

The second data set, referred to as MUV, corresponds to a subset of 26 eyes from a long-term and ongoing observational study of drusen development at the Department of Ophthalmology, Medical University of Vienna (MedUni Wien) of participants (85% female with a mean age of 74 ± 11 years) as reported previously.^4^ The MUV data set contains baseline OCT scans of participant eyes with drusen and no late AMD.

All participants gave informed consent prior to inclusion in the respective studies. This retrospective analysis was approved by the Ethics Committee at MedUni Wien (EK Nr: 1246/2016). All study procedures were conducted in accordance with the Declaration of Helsinki, and all the personal data were pseudonymized.

#### OPL Subsidence Grading and Annotation

**Figure 1.**
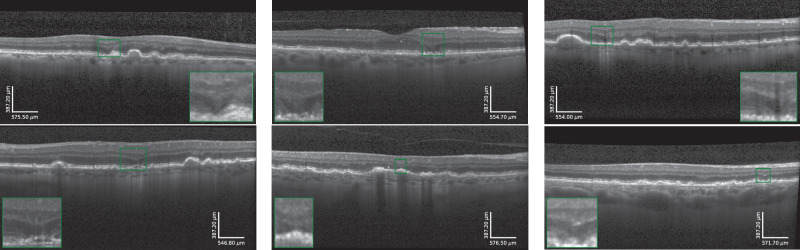
Representative examples of manually annotated OPL subsidence (*green box* with magnification) from the data sets used in this study.

In both data sets, the OCTs were acquired with a Spectralis HRA + OCT (Heidelberg Engineering, Heidelberg, Germany). Images covered an en face field of view of 20° × 20° with 49 B-scans containing 512 to 1024 × 496 pixels. Only the OCTs before the onset of late AMD (macular neovascularization or cRORA) were included in this study, with late AMD graded by a team of retinal experts at MedUni Wien (S.R. and C.G.). OPL subsidences were manually graded and annotated by a team of retinal experts (S.R. and G.S.R.) at MedUni Wien by denoting all the A-scans within an OCT volume where the OPL subsidence was deemed present. Specifically, the subsidence of the posterior and anterior boundary of OPL (i.e., the OPL/outer nuclear layer (ONL) and INL/OPL junction) had to be observed. Examples of representative annotations are shown in Figure 1. Examples of representative annotations are shown in Figure 1.

In LEAD, all the included OCTs were graded for the presence of OPL subsidence, and the subsidence was annotated whenever present, except the eyes with subsidence that did not develop cRORA (9% of the eyes), where only one OCT volume per subsidence was included and the subsidence annotated. In MUV, the baseline OCTs of 26 patients (selected sequentially by pseudo-ID) were graded for the presence of subsidence, and the subsidence was annotated whenever present. The characteristics of the two annotated OCT data sets are summarized in the Table.

**Table.** Characteristics of the LEAD and MUV Annotated Subsets Used for Developing and Externally Testing the Automated INL and/or OPL Subsidence Detection Method

	Without OPL Subsidence	With OPL Subsidence	Total
LEAD			
Subjects	94	46	140
Occurrence	Both eyes: 94	Just one eye: 26^a^/both eyes: 20	140
Female/male	73/21	35/11	108/32
Age, y	69 ± 8/70 ± 7	70 ± 9/69 ± 2	70 ± 8
Eyes	213^b^	66	279
Volumes	1599	130	1729
MUV			
Subjects	20	6	26
Female/male	16/4	6/0	22/4
Age, y	73 ± 12/69 ± 6	79 ± 6/−	74 ± 11
Eyes	20	6	26
Volumes	24	6	30

a Includes a patient for which only one eye was annotated due to cRORA onset at baseline.

b The number of eyes without OPL subsidence corresponds to the number of nonaffected eyes (2 × 94) plus the number of eyes without subsidence from patients in which only one eye is affected (26−1).

### Deep Learning Model for Automated Subsidence Detection

**Figure 2.**
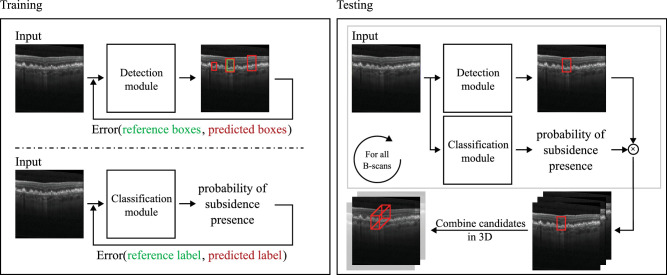
Training and testing pipelines for the DM and the CM.

The system presented in this study receives as input a retinal OCT volume and outputs (1) a set of 3D bounding boxes enclosing OPL subsidence candidates and their corresponding confidence and (2) a volume-wise OPL subsidence confidence, corresponding to the maximum confidence of all the bounding boxes. For this purpose, our method is composed of two deep learning–based modules, both operating at the B-scan level. First, a detection module (DM) is responsible for generating bounding boxes for subsidence candidates with high sensitivity. A classification module (CM) is then used to reduce the number of FP detections. In particular, CM infers the likelihood of a given B-scan containing OPL subsidence. For each B-scan, the likelihood of each candidate from DM is then multiplied by the score from CM (i.e., the final candidate confidence will only be high when both CM and DM agree on the prediction). To provide extra spatial information to the system, the input is a three-channel image composed of the current B-scan of interest and the two nearest neighboring B-scans (one before and one after). For the first and last B-scans of the volume, we repeat one of the B-scans to complete the image. At test time, all B-scans are processed individually, and overlapping OPL subsidence candidates across neighboring slices are merged. A summary of the training and testing pipelines is shown in Figure 2.

#### Classification Module

The CM is based on the ConvNeXt architecture^26^ pretrained on the ImageNet.^27^ ConvNeXt is a recently introduced convolutional neural network (CNN), shown to be state of the art in natural image classification. In our approach, the network receives a B-scan resized to 224 × 224 pixels as input and has a single output neuron with a sigmoid activation to score subsidence presence ∈ [0*,* 1]. Only volumes with at least one subsidence case were used for training and validation. We use a class-balanced training scheme by using batches with the same number of B-scans with and without subsidence. The optimizer was Adam^28^ with a learning rate of 10^−4^ and data were augmented using translations, rotations, scaling, and intensity alterations. The model was trained for 1000 epochs with 16 samples per batch, and the model with the best validation set performance was selected.

#### Detection Module

The DM is based on the Mask R-CNN,^23^ a CNN used for automated object detection and segmentation. In particular, an ImageNet pretrained ConvNeXt is used for extracting features, which then serve as input for an object detection block. The object detection block is composed of a region proposal network (RPN), which is responsible for selecting regions inside the image that potentially contain OPL subsidence. The features from the RPN-proposed regions are then extracted and resized to a standard dimension via the region of interest align (RoIAlign) block. The output of the RoIAlign is then used by an object detection head to dimension and score the found bounding boxes. DM receives as input a B-scan resized to 256 × 256 pixels, and only B-scans with at least one subsidence case were used for training. The optimizer was AdamW^29^ with an initial learning rate of 10^−4^ and an epoch-wise decay rate of 0.95. The model was trained for 100 epochs with 16 samples per batch and the model was kept.

#### Prediction Module

The final detections are obtained by merging the results of the DM and CM modules for all B-scans, as shown in Figure 2. In detail, for each B-scan, the scores of the candidates predicted by DM are multiplied by the scan-wise score from CM. Overlapping candidates are then combined across neighboring B-scans to produce cuboids enclosing potential OPL subsidence cases. For each two adjacent B-scans, the overlap between each possible pair of bounding boxes is measured using the intersection over union (IoU) and considered part of the same lesion prediction if IoU >0.5. For each group of candidates, the final prediction cuboid corresponds to the minimum possible volume that encloses all candidates. The confidence of each of these 3D subsidence candidates is taken to be equal to the maximum confidence of the candidates that it encloses.

### Statistical Analysis and Evaluation Metrics

In our study, detected OPL subsidence cases are considered TP if they spatially match a manual annotation, FP if a candidate does not overlap with any annotation, and false negative (FN) if a manual annotation has no corresponding candidate. For classification tasks, a case is TP if both prediction and the manual reference indicate OPL subsidence presence, true negative (TN) if both indicate the absence of this type of lesion, FP if the system predicts the subsidence presence on a case without it, and FN if the opposite occurs.

The following base metrics are used: (1) true-positive rate (or recall), TP/(TP + FN) ∈ [0, 1], corresponds to the proportion of positive cases correctly identified as such; (2) false-positive rate (or specificity), FP/(FP + TN) ∈ [0, 1], is the proportion of cases wrongly identified as being negative; and (3) precision, TP/(TP + FP) ∈ [0, 1], is the ratio of positively identified cases that are actually positive.

These metrics allow us to draw performance curves. In particular, we consider the (1) free-response receiver operating characteristic (FROC) curve, which plots the true-positive rate as a function of the number of FP detections resulting from different thresholds on the predicted confidence; (2) receiver operating characteristic (ROC) curve, which plots the true-positive rate as a function of the false-positive rate, obtained by varying the decision threshold on the predicted confidence; and (3) precision-recall (PR) curve, which shows the precision as a function of the true-positive rate, also obtained by varying the decision threshold. The PR curve is of particular interest for highly imbalanced screening settings, as in this study, since it focuses only on the detection of the positive cases. The ROC curve can be summarized by its area under the curve (AUC) ∈ [0, 1], with AUC = 0.5 indicating a random predictor and AUC = 1 a perfect classifier. The PR curve can be summarized with the average precision (AP), the weighted mean of the precisions achieved at each threshold, with the increase in recall from the previous threshold used as the weight. We consider the PR curve baseline as P/(P + N), where P and N are the number of positive and negative cases, respectively.

The association between the baseline presence or development of OPL subsidence and the development of the endpoint of GA as assessed on color funds photography was assessed on the LEAD data set, analogously as done previously for iRORA and nGA.^7^^,^^9^ An univariable Cox proportional hazard model was used both with and without adjusting for age, gender, and smoking status at baseline. Confidence intervals (CI) for the hazard ratio (HR) were computed using bootstrap resampling (*n* = 1000 resamples). Finally, the unadjusted proportion of variation in the time to develop GA explained (*R*^2^) by the presence or development of OPL subsidence was calculated. Analyses were conducted using Stata/IC software 14.2 (StataCorp, College Station, TX, USA).

## Results

Characteristics of the data sets available for development and external testing are summarized in the Table. In the LEAD data set, eyes with prevalent or incident OPL subsidence were associated with a significant increase rate of GA development (unadjusted HR, 28.9; 95% CI, 6.1–113.7; *P* < 0.001; adjusted HR, 30.9; 95% CI, 6.3–150.5; *P* < 0.001). The proportion of variance explained in the time to GA development by the OPL subsidence itself was 78% (*R*^2^). This confirms the hypothesis that the subsidence defined here is a highly predictive risk factor for progression to GA.

Evaluation of the subsidence detection on the LEAD data set was conducted using stratified 10-fold cross-validation at the participant level while maintaining the proportion of those with and without OPL subsidence. The MUV data set has been used as an independent external data set from the one where the model was developed. There, we assess each of the models trained on the folds from the LEAD data set. The method was successfully capable of generating candidates for all cases in both data sets. All the experiments were performed using Python 3.9 and PyTorch 1.12.1, on a workstation with an Intel Core i7-10700K CPU and NVIDIA RTX3080 GPU and using a server with AMD EPYC 7302, 16-core CPU and NVIDIA RTX A6000 48 GB GPU. Using the workstation, initial predictions for an OCT volume can be obtained in less than 1.5 seconds.

### OCT Volume Classification

In this experiment, we assessed if the system can be used for identifying OCT volumes that contain at least one OPL subsidence case. For that, each OCT volume is assigned a confidence corresponding to the maximum confidence of the predicted candidates. We evaluate the performance for all cases on all folds using the ROC and PR curves and their corresponding AUC. This evaluation is conducted at (1) OCT volume level; (2) eye level, where each eye is attributed the maximum corresponding volume-level confidence; and (3) participant level, with the scoring corresponding to the maximum confidence across all the volumes of the two eyes. Eye- and participant-level OPL subsidence detection is of particular interest for building data sets/performing studies from retrospective data, allowing to find participants with potential OPL subsidence while significantly reducing the need for manual assessment.

**Figure 3.**
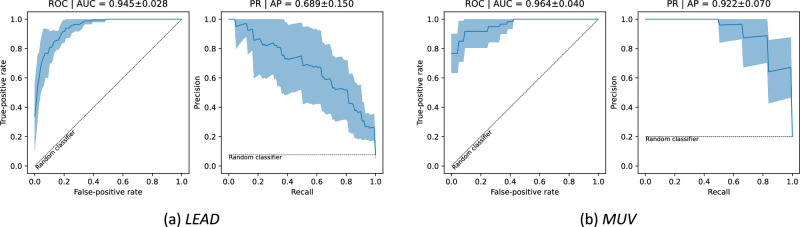
Volume-wise ROC and PR curves and corresponding AUC values.

The ROC and PR performance curves for OPL subsidence classification in the LEAD data set for the OCT volume level are shown in Figure 3. Refer to Appendix A for the eye- and participant-level performances and to Appendix B for the module-wise B-scan and volume-level performances. We also report the volume-level performance for MUV. The high ROC AUC and PR AP values indicate that the system is capable of discriminating between cases with and without OPL subsidence at all volume, eye, and participant levels. The performance reported in Figure 3b further corroborates the robustness of the method, as it maintains its performance on the external data set.

### Subsidence Detection

**Figure 4.**
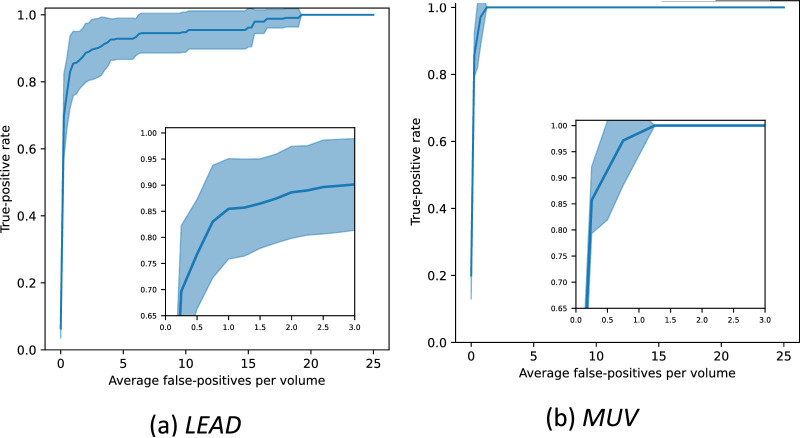
FROC curve for OPL subsidence detection for the two data sets. The *line* corresponds to the average FROC curve across all folds, and the *shaded area* is the corresponding standard deviation.

Complementarily to the volume-level subsidence detection, we also assessed the capability of the system to properly localize and score OPL subsidence cases. All volumes of each test fold were checked to determine if at least one predicted candidate overlapped with each manually annotated OPL subsidence. An OPL subsidence was considered detected if at least one candidate had IoU ≥0*.*25. If there were multiple candidates for the same OPL subsidence, we only considered the one with the highest confidence (the remaining are counted neither TP nor FP). We evaluated the detection performance for each fold considering all OPL subsidence cases independently, to confirm the reliability of the automated detection. The results of this experiment were evaluated via a FROC curve, shown in Figure 4.

The average sensitivity for one, two, and three FPs per volume for the LEAD data set (Fig. 4a) was 0*.*85 ± 0*.*10, 0*.*89 ± 0*.*09 and 0*.*90 ± 0*.*09, respectively. In the MUV data set (Fig. 4b), for the same FPs per volume, we achieved sensitivities greater than 0.99. Results suggest that our model can be reliably used as a screening tool, detecting most OPL subsidences in the correct location with a small number of FPs per scan.

**Figure 5.**
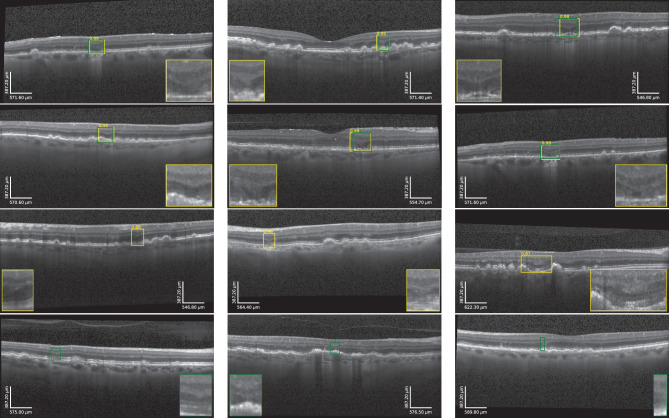
Examples of automated OPL subsidence detection in the LEAD data set agreeing with manual annotations (rows 1–2), not agreeing with manual annotations (row 3), and missed OPL subsidences (row 4). Manual annotations are in *green* and automated detections are color-scaled from *red* to *yellow* as a function of the corresponding prediction confidence.

Qualitative OPL subsidence detection examples are shown in Figure 5. As illustrated, the system is capable of detecting OPL subsidence cases with different morphologic manifestations (rows 1–2). Highly confident automated detections that did not match manual annotations (row 3) largely corresponded to cases that share partial morphologic manifestations with OPL subsidences. Missed cases commonly correspond to very subtle subsidences, as illustrated in row 4.

## Discussion

Detecting early biomarkers of retinal atrophy is expected to play an increasingly important role as surrogate endpoints of GA and for enriching high-risk intermediate AMD cohorts. We have reported here that OPL subsidence is a high-risk early biomarker for the risk of progression to GA, and as such, its identification is of value. When the LEAD cohort was reanalyzed for this research, the presence of OPL subsidence alone was associated with a 30-fold increased risk of GA development, which was higher than the presence of iRORA^5^ but lower than the presence of nGA in the same cohort.^9^ Identification of OPL subsidence would be of value either during routine monitoring visits, when mining large repositories of retrospective OCT scans for potential inclusion in early intervention studies, or in reading centers tasked to identify early atrophic biomarkers as endpoints in early intervention trials. In these scenarios, automated AI-based solutions would be very useful. While other factors associated with increased risk of AMD progression, such as subretinal drusenoid deposits or hyperreflective foci, are not as specifically related to an area that will subsequently develop GA, OPL subsidence is a location-specific biomarker. Therefore, it highlights certain areas that are at high risk of becoming atrophic. Identifying these areas will be useful when considering the management of individual patients, in whom, for example, the proximity to the fovea would be valuable information to take into consideration.

Deep learning has already shown potential for a wide range of medical-related tasks, including cancer screening, brain assessment, and cardiovascular diseases.^30^^–^^33^ Indeed, ophthalmology is one of the fields where deep learning has been most successfully explored.^34^ For instance, studies have shown that deep learning can enable several automated screening tasks that require the analysis of eye fundus photography and fundus autofluorescence images, including AMD, diabetic retinopathy, and glaucoma.^35^^–^^39^

With this in mind, we developed a deep learning–based method for automated OPL subsidence detection in OCT images. We handle the task using an object detection approach in contrast to the more common pixel-wise segmentation available in the literature for other retinal OCT biomarkers. In fact, OPL subsidence, unlike other biomarkers (especially those fluid related), is harder to quantify volumetrically as it is not a formation of novel features on the OCT but rather a sudden change in the shape of the retinal layers. We argue that this makes a region-of-interest characterization (i.e., centroid and bounding box) more appropriate compared to a pixel-level segmentation or an analysis of retinal layer segmentation. In addition, we opted for a two-step approach, with an initial high-sensitivity candidate detection (DM) followed by an FP reduction using a different model (CM). Although this increases the overall computational burden of the approach to approximately 1.5 seconds per OCT volume, combining the prediction of multiple models is known to improve deep learning–based systems’ robustness,^40^ which is essential for medical applications.

The results show that our method can be used to confidently classify volumes, eyes, and subjects according to the presence of OPL subsidence (Fig. 3, Appendix A). We have also shown that the system was capable of detecting most OPL subsidence cases at the correct location with a very limited number of FP detections (Fig. 4). The high detection performance on both the development and the external data set, together with the diversity of detected subsidence morphologies, as illustrated in Figure 5, confirms that both DM and CM properly learned the expected morphologic manifestations of OPL subsidences (Appendix B). Importantly, the consequent robustness and reliability allow our approach to be used for a fast and objective interpretation of OCTs contributing to reducing the manual workload and facilitating the detection of this important biomarker. In an increasingly data-driven field, as is ophthalmology, such systems can contribute to creating large prospective cohorts and curating large retrospective study cohorts in a rapid fashion by automatically preselecting cases with or without the presence of a biomarker of interest, in this case the OPL subsidence, as required by the study design.

The OPL subsidence detected in this study is an isolated imaging feature and therefore different from the original definition of nGA where both anterior and posterior boundaries of the INL and the OPL needed to show subsidence, or there could be the sole presence of a hyporeflective wedge-shaped band within Henle's nerve fiber layer or both. A comparison of time-to-event analyses confirms that the subsidence of the OPL studied here was a significant risk factor for progression in LEAD with an HR of 30, although it was considerably less than that for nGA with an HR of 78 in the same cohort. Future work could be to train a model to detect the features that fully describe nGA—the subsidence of both the OPL and INL and/or the hyporeflective wedge-shaped band within HFL. Nevertheless, we expect that the training and development paradigm proposed here would have similar success if the full morphologic manifestations that define nGA had been the prediction target of interest.

The study has several limitations. Despite its high sensitivity, the proposed system still fails at detecting very subtle OPL subsidences, as shown in Figure 5 (row 4), but future work will be needed to understand the importance of missing such subtle subsidences. At the same time, these subtle subsidences are expected to have higher interreader variability, and although manual gradings were performed by experienced retinal experts, minor discrepancies cannot be ruled out in those cases. With this in mind, conducting such interreader variability studies would allow a better assessment of the performance of the system. Likewise, this study focuses only on scans acquired with Spectralis OCT devices. As known, DNN-based systems are very sensitive to changes in acquisition settings, and thus there is no guarantee that our findings extend to other devices. Further efforts should thus focus on identifying preprocessing and training strategies that allow for a better generalization of these types of approaches. Furthermore, our method was not trained for and the study does not evaluate the performance of our system for OCT volumes with late AMD (i.e., neovascular AMD and the presence of cRORA). Similarly, our training data are vastly composed of people of Anglo-Saxon descent, and although our study suggests that the model has generalization capability, there is no guarantee that its performance will be the same for different ethnicities and demographic compositions. In the same line, our external independent data set, MUV, has a relatively small sample size, weakening our understanding of how well the system is generalizable to other cohorts. Future work should hence focus on extending both development and test data sets.

In conclusion, we present a DNN-based approach for the automatic detection of OPL subsidence in retinal OCT B-scans. Our extensive quantitative and qualitative evaluation shows that the proposed approach is capable of efficiently detecting OPL subsidence, an early biomarker for the risk of progression to GA in the setting of AMD. In particular, it can be used as a tool to help understand risk of AMD progression and for mining large-scale OCT data sets for the presence of OPL subsidence, to enrich iAMD cohorts for inclusion in interventional trials aiming to slow progression early in the disease process. Finally, such automated systems have a great value in reading centers aiming to identify the early signs of cell loss and atrophy, which can be used as endpoints for early intervention trials in AMD, greatly facilitating their design and implementation.

## Figures and Tables

**Figure A1.**
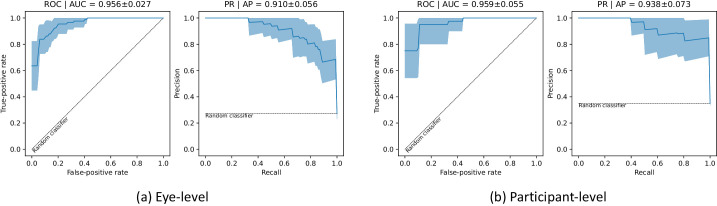
Performance of the OPL subsidence presence classifier on the *LEAD* dataset at the eye- and participant-level. Both eye (a) and participant-wise (b) receiver operating characteristic (ROC) and precision-recall (PR) curves, and corresponding area under the curve (AUC/AP) values, are shown.

**Figure A2.**
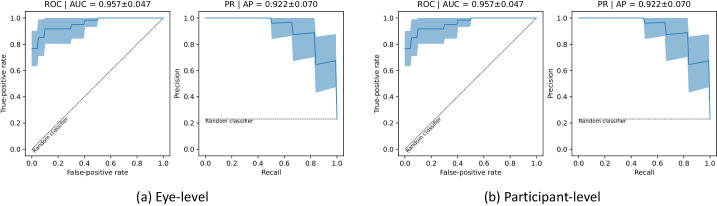
Performance of the OPL subsidence presence classifier on the *MUV* dataset at the eye- and participant-level. Both eye (a) and participant-wise (b) receiver operating characteristic (ROC) and precision-recall (PR) curves, and corresponding area under the curve (AUC/AP) values, are shown.

**Figure B1.**
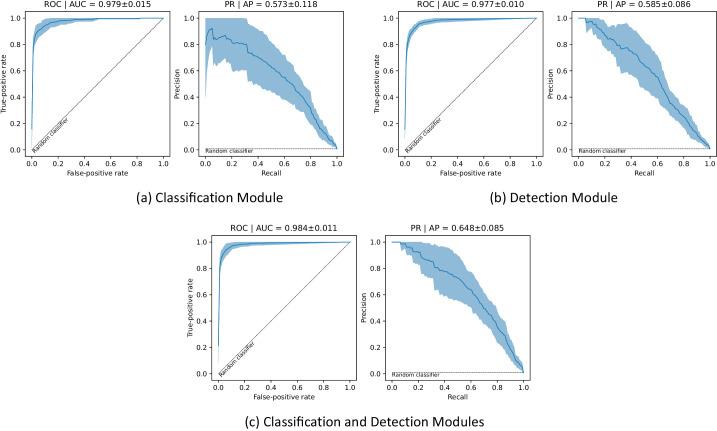
B-scan level performance of the OPL subsidence presence classifier on the *LEAD* dataset: (a) Classification Module only; (b) Detection Module only and (c) Classification and Detection Module (proposed system).

**Figure B2.**
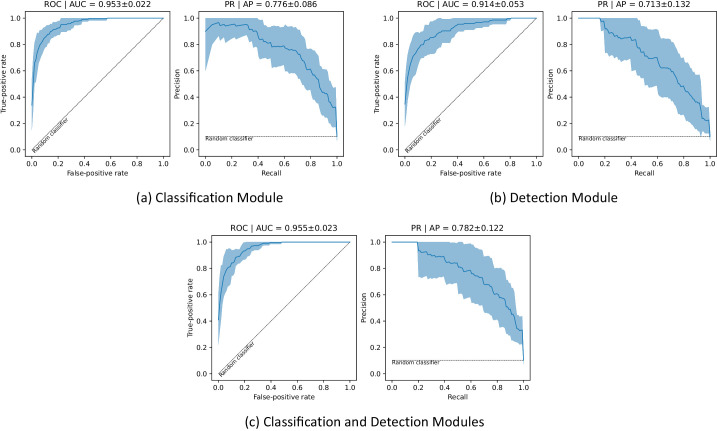
Volume level performance of the OPL subsidence presence classifier on the *LEAD* dataset: (a) Classification Module only; (b) Detection Module only and (c) Classification and Detection Module (proposed system).

**Figure B3.**
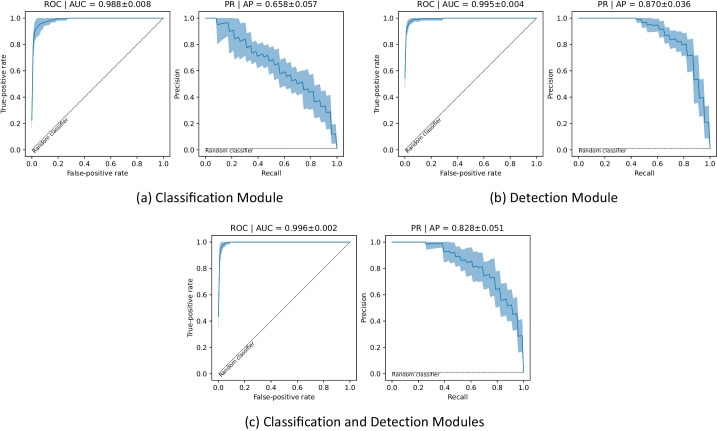
B-scan level performance of the OPL subsidence presence classifier on the *MUV* dataset: (a) Classification Module only; (b) Detection Module only and (c) Classification and Deteciotion Module (proposed system).

**Figure B4.**
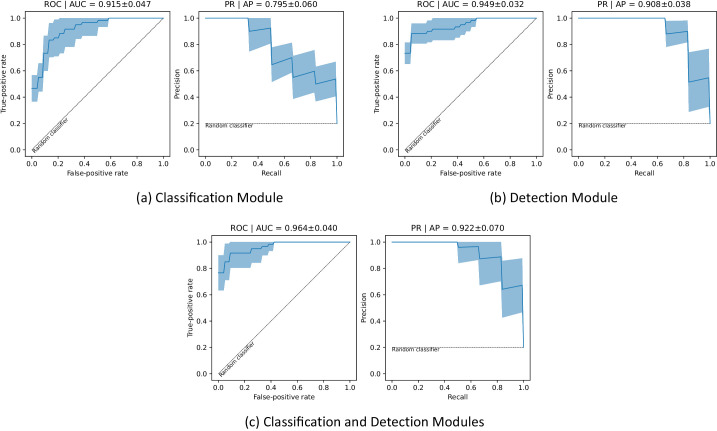
Volume level performance of the OPL subsidence presence classifier on the *MUV* dataset: (a) Classification Module only; (b) Detection Module only and (c) Classification and Detection Module (proposed system).

## Appendix B

**Table B1.** B-Scan and Volume Level Area Under the Receiver Operating Characteristic (ROC) Curve (AUC) and Average Precision (AP) for the *LEAD* and *MUV* Datasets

	Modules		LEAD		MUV	
Level	CM	DM	AUC	AP	AUC	AP
B-scan	✓		0.979 ± 0.015	0.573 ± 0.118	0.988 ± 0.008	0.658 ± 0.057
		✓	0.977 ± 0.010	0.585 ± 0.086	0.995 ± 0.004	0.870 ± 0.036
	✓	✓	0.984 ± 0.011	0.648 ± 0.085	0.996 ± 0.002	0.828 ± 0.051
Volume	✓		0.953 ± 0.022	0.776 ± 0.086	0.915 ± 0.047	0.795 ± 0.060
		✓	0.914 ± 0.053	0.713 ± 0.132	0.949 ± 0.032	0.908 ± 0.038
	✓	✓	0.955 ± 0.023	0.782 ± 0.122	0.964 ± 0.040	0.922 ± 0.070
